# The material consumptive: domesticating the tuberculosis patient in Edwardian England

**DOI:** 10.1016/j.jhg.2012.12.007

**Published:** 2013-10

**Authors:** Graham Mooney

**Affiliations:** Institute of the History of Medicine, Johns Hopkins University, 1900 East Monument Street, Baltimore, MD 21205, USA

**Keywords:** Tuberculosis, Consumption, Home, Domestic space, Disinfection, Sanatorium, Self-help, Preventive therapy, Sheffield

## Abstract

The proliferation of general and specialist hospitals, lunatic asylums, and workhouse infirmaries in the nineteenth century challenged the popular perception of the home as a suitable site of health care. Amidst the emergence of yet another type of institution, the tuberculosis sanatorium, tuberculosis control in the Edwardian period was re-sited and re-scaled to accommodate what might be termed a ‘preventive therapy’ of domestic space. Three interlinked perspectives demonstrate why and how this happened. First, I explore the role of the national and local state in legitimating domestic space as a scale and a site for the regulation of tuberculosis patients and prevention of the disease. Second, I investigate how tuberculosis self-help manuals promoted a technology of the self that was founded largely on the principles of sanatorium therapy but was necessarily reconfigured to reflect the social relations of domestic space. Third, I assess the marketing of consumer goods to the domiciled tuberculosis sufferer through the pages of the *British Journal of Tuberculosis*. It is suggested that a common tubercular ‘language’ of material consumption was fashioned in order to normalise the accumulation of possessions for use in the home. These arguments are situated in relation to recent historical research on material culture and identity at the turn of the twentieth century, which has stressed the cultivation of individuality and that the right sort of possessions appropriately arranged in domestic space signified well-regulated morality.

## Highlights

► Show how and why domestic space was produced as a site and scale of tuberculosis therapy. ► Examine how the regimen of tuberculosis sanatoria was adapted to home treatment. ► Explore how home tuberculosis therapy was linked to mass consumption and self-help. ► Reveal the moral components of domestic tuberculosis therapy.

The nineteenth century witnessed an unprecedented transformation in the locus of care for the sick in Britain. General and specialist hospitals, insane asylums and workhouse infirmaries warehoused ever greater numbers of ill people. The perception that the home was the natural space in which to tend to the unwell was destabilised by this shift towards the institutionalisation of health care. Another type of institution was the tuberculosis sanatorium. Sanatoria emerged in Europe and the United States towards the end of the nineteenth century as part of an approach to controlling the disease that also included the dissemination of behavioural advice through health education and the regulation of meat and dairy products. These latter interventions were amongst the first public health policies to address the problem of tuberculosis directly and were related to the idea of tuberculosis as a ‘social disease’ that dominated policies in Britain and its colonies.^1^

Whilst we know a great deal about the cultural and social significance of sanatoria in the Edwardian period, it nevertheless remained the case that the vast majority of tuberculosis patients were not institutionalised. The dawning realisation that it was unrealistic to hospitalise the mass of tuberculosis sufferers prompted a reformulation of regulation that focused efforts on the home as a viable site of intervention. The domestication of tuberculosis was achieved by moulding elements of existing public health policy with components of the sanatorium regimen into a kind of domesticated ‘preventive therapy’ for tuberculosis.^2^ This meant deployment of tuberculosis surveillance and disinfection, but without the threat of mandatory hospitalisation. The sanatorium formula of rest, exercise, and diet was adapted to the domestic environment and promoted a ‘technology of the self’ through the social relations of domestic space.^3^ Equally significant was the fashioning of a tubercular ‘language’ of material consumption that promoted the accumulation of possessions for use in the home. The *British Journal of Tuberculosis* (*BJTB*) in particular endorsed and promoted a vast array of everyday objects and appliances—baths, reclining chairs, bed rests, reading stands and many, many more—that bridged the therapeutic divide between institutional and domestic space.^4^ By encouraging a culture of possession, tubercular patients were drawn into the realm of mass consumerism that normalised their identity.

As a result of these multiple strategies, tuberculosis control was re-scaled and re-sited from the sanatorium to the home. The national and local state came to share the regulatory site of the home with alternate, but complementary, bureaucracies of power. Some experts outside formal government were armed with medical knowledge and hygienic ideas; others deployed marketing strategies, advertising skills and the language of selling.^5^ The specifics of this sort of re-scaling and re-siting have largely been overlooked by historians, though the form and idea of ‘home’ and ‘domesticity’ have been much debated, particularly for the Victorian period. It is important, then to resist naturalising scale as a pre-existing component of spatiality that historical actors operate within or pass between and through. For the purposes of this paper, sensitivity to the production of scale (for example, national, local, urban, rural, domestic) and site (such as sanatorium, home) helps disclose the underlying scope of moral regulation inherent in the tactics of tuberculosis control at the end of the nineteenth century and the beginning of the twentieth. For example, the cultivation of individuality and the right sort of possessions appropriately arranged in domestic space signified a well-regulated morality.^6^ In the case of tuberculosis, a multitude of consumer goods were marketed to the domiciled sufferer as the material expression of a morality that conditioned the behaviour of patients. They nurtured domesticity, homely pastimes and healthy pursuits that were as much an antidote to morally corrupt activities as they were crucial to therapeutic success. Moral regulation here concerns how ‘morality’—that is, normative judgements about what is wrong or bad conduct across a broad range of behaviours^7^—was channelled through disease and health, at a time when wider notions of subjectivity were undergoing change.

The paper addresses these issues in four main sections. The first two parts outline the ways in which public health administrations re-scaled and re-sited tuberculosis strategies as ‘domestic’ and how these strategies can be understood in terms of subjectivity. In this respect, the discourses and practices around what was one of the most pressing health issues of the early Edwardian period are used to build on the recent work in historical geography by Sallie Marston, Alison Blunt and others on the political context of everyday and mundane activities. It is stressed that the production of ‘domestic’ as a geographical scale and the management of ‘home’ as a site of risk and opportunity reveals much about the structure and exercise of power.^8^ The third and fourth sections examine the shifting terrain of subjectivity in the early twentieth century (from ‘character’ to ‘personality’) through tuberculosis self-help manuals that were written for domiciled patients, and the materialisation of domestic preventive therapy via analysis of a section in the *BJTB* which promoted products for tuberculosis patients. One of the key points to emerge is the extent to which the market for consumer goods operated as a crucial arena for moral regulation through health.

## Subjectivity, site and scale in British public health

The Edwardian period witnessed a shift in subjectivity from one that was rooted in ‘character’ shifted to one based on ‘personality’. The former pointed to conformity with a set of public virtues that ‘comprised a citizen's moral constitution’, while the latter was based around the formation of the unique self, associated with the tantalising quest for individuality.^9^ Although this sort of narrative about subjectivity is undoubtedly over-generalised, its broad contours can be meshed with an account of the politics of public health in the nineteenth century in order to understand the process of how and why the ‘domestic’ came to be reconstituted as a necessary scale of tuberculosis therapy.^10^

Scalar issues were significant in public health in the early Victorian period, when much activity centred on ambitious infrastructural projects that sought to remediate the detrimental environmental aspects of urban industrial growth.^11^ Victorians struggled with the conceptual and geometric scale of these sanitary schemes.^12^ The political tussle over sanitary measures was essentially a contest made through hierarchical scale—the national centre and the local province.^13^ Yet Victorian sanitary reform was replete with tacit moral assumptions about domestic space, issues, and relations.^14^ Connections between the intimate space of the home and the public space of the environment were several and layered.^15^ Sanitarians argued that the degradation of urban places fostered immoral habits and vice. An environmentally-based public health offered the chance to create the appropriate conditions under which individuals (or more precisely, voting men) might achieve *domestic* propriety.^16^ National debate about the electoral franchise between the 1830s and the 1860s constantly circled this issue and qualification for the vote in the 1867 Reform Act solidified the ideal of the domestic man with ‘a house, a wife, children, furniture and the habit of obeying the law’.^17^

Historians Patrick Joyce and Chris Otter have argued that drains, sewers and water mains were material expressions of liberalism that mediated the freedom of the governed to act rationally in public and private space.^18^ From the 1870s, however, management of the physical environment in order to help create favourable domestic circumstances was complemented by a set of public health interventions that explicitly interfered with the social relations of individuals inside the home itself.^19^ The notification of infectious diseases (under which private family doctors were paid by the local health authority to report infectious cases to its medical officer), the isolation of the infected, contact tracing, and the destruction or neutralisation of biological threats with disinfection, comprised a set of surveillance practices that disrupted the intimate channels of disease transmission between humans, even if those channels were barely understood.^20^ Such techniques—which excluded tuberculosis and focused mainly on childhood infections such as scarlet fever and diphtheria—were adopted incrementally in the late nineteenth century, though they were most fully realised in towns and cities that had the political drive and financial wherewithal to implement them. These interventions preceded the emergence and acceptance of germ theory, though they were retrospectively endorsed and refined by the more precise knowledge of disease transmission furnished by bacteriological research.^21^ Infectious disease surveillance characterised a more individualised approach to public health at the turn of the twentieth century that mirrored and was constitutive of the broader shift to subjective individuality.

Establishing and preserving a biologically risk-free home was a barometer of hygienic citizenship. But the administrative structures, policies and techniques of infectious disease surveillance in Britain were poly-scalar and poly-sited. Infectious patients were medically managed in multiple sites, such as isolation hospitals or separate wards in general hospitals and poor law infirmaries. A lack of available beds restricted hospital admissions in many places, but the Sanitary Act of 1866 declared that patients would be hospitalised if their home did not have ‘proper lodging or accommodation, or lodged in a room occupied by more than one family’. The definition of ‘proper’ was left to the discretion of local health authorities and interpretation of the law implicitly preserved the privacy of middle-class households—poor families were more likely to lodge together and rarely had enough space at home to demarcate a sickroom.

Meanwhile, national government trod a cautious scalar path: public health policy debates were driven by the conflicting demands of international/national, local/national, urban/rural, and individual/community constituencies. As a consequence, much national public health legislation was permissive rather than compulsory. For example, while some districts adopted infectious disease notification through local acts of parliament, it took more than two decades for central government to impose it on all local authorities. Discourse on infectious disease surveillance was strongly influenced by a scalar argument: it was pointless to have city-wide policies to regulate urban residents yet not subject the inhabitants of the surrounding rural hinterlands to the same sorts of restrictions.^22^ The solution to this uneven and biologically dangerous geography was to mandate infectious disease notification across the whole nation, which finally happened in 1899.

The contested status of tuberculosis as a communicable disease provided an interesting dilemma for advocates of infectious disease surveillance. The position of tuberculosis as the biggest (though declining) killer provoked multiple explanations as to its proximate causes. As Michael Worboys has shown, between 1880 and 1930, sanitary conditions (including housing), person-to-person contact, behaviour and lifestyle, inherited susceptibility, and herd immunity were all put forward as possible reasons for why tuberculosis was declining.^23^ To varying degrees, components of the first three influenced the way in which domestic space was articulated as a site and scale of intervention to regulate patients and prevent the disease.

## Disinfection, tuberculosis, and mobilisation of the domestic

The characterisation of a risk-abundant domestic space was fairly commonplace by the 1860s.^24^ The mid-1870s and early-1880s were replete with warnings about the evils of dust. Particular ire was reserved for the dust-retaining properties of carpets, curtains and ornate hangings.^25^ In his address on ‘Domestic Health’ delivered to the annual meeting of the Sanitary Institute in Brighton, the well-known public health activist Alfred Carpenter said in 1881 that, ‘there is scarcely a house in the kingdom in which excreta are not to some extent retained’. ‘Excreta’ here means not just drain flush, but all forms of bodily seepage that never made it beyond the confines of the home:

The most civilised and luxurious home is, in some cases, carefully prepared for the cultivation of disease-germs or factors, if they come into our midst: carpets, curtains, and comforts of all kinds retain the débris from our skins and our pulmonary membranes; the excreta from our sweat-glands are allowed to settle upon our uncleaned windows, out-of-the-way cornices, useless ledges, and so-called architectural or upholstering ornaments.^26^

By shifting the geography of risk ineluctably inwards, public health forced itself into the space of the mundane, the everyday and the domestic.^27^ There was, of course, a massive popular literature on design and decoration of the domestic interior and this was directed predominantly at women as the moral guardians of the home.^28^

After the 1866 Sanitary Act, householders' own hygienic efforts were supplemented by municipal disinfecting activities that entered domestic space. From the late 1870s in urban Britain, bacteriological experiments and technological know-how were harnessed to create highly mobile, city-wide systems of disinfection that deployed pressurised steam and a cocktail of chemicals to rid peoples' homes, clothes and belongings of dangerous microbes. Though municipal disinfection of homes was geographically variable, incomplete, and deployed both ‘old’ (fumigation) and ‘new’ (disinfecting) methods, public health officers were extremely confident by the early 1900s that they could eliminate most biological threats from the domestic environment.^29^

Much of this confidence can be accredited to bacteriological research in the 1890s on tubercular dust (that is, the dried sputum of the tuberculosis patient). This research was readily grafted onto existing anxieties about the accumulation of dust and dirt in the over-adorned Victorian domestic space.^30^ It was a common trope of hygienists that dust was a ‘carrier’ or ‘common conveyer’ of tuberculosis and that the tuberculosis bacillus ‘lingers long in the dust of rooms, inhabited by careless tuberculous subjects’.^31^ Eradicating this ‘matter out of place’ became a hygienic duty and conditioning the behaviour of these reckless individuals became a central plank of effective anti-tuberculosis campaigns.^32^

Despite clarification from the early-1880s that tuberculosis was indeed a communicable disease, a prolonged period of institutional sequestration was not a viable option for the vast majority of sufferers due to the potential loss of earnings and social functioning. Domestic space had to be the main therapeutic site. If home-based interventions were to have any impact, as many patients as possible needed to be reached and as many risks as possible nullified. Under this rationale, the notification of tuberculosis was the only realistic way forward and a number of voluntary schemes were developed.^33^ The first, in Brighton in 1899, was quickly copied elsewhere and by 1904 eleven other towns encouraged GPs to voluntarily inform the sanitary authority of tuberculosis cases coming under their care.^34^

Public health officials and tuberculosis campaigners complained that voluntary notification only revealed a limited number of sufferers; it was thought that Sheffield's scheme routinely missed between 40% and 50% of all cases in the city.^35^ Sheffield became the first city to win parliamentary approval for compulsory tuberculosis notification in 1903. Up to this date, the Local Government Board (LGB) routinely opposed proposals from other towns, but public health administrators from Sheffield worked hard behind the scenes to alter the LGB's position.^36^ In May 1903 negotiations took place with the LGB's medical officers (one of whom, Dr Theodore Thomson, just happened to be a former Medical Officer of Health for Sheffield^37^) to draft a law that was acceptable to central government.^38^

Sheffield's representatives argued that the public themselves did not see notification as problematic and witnesses attested that ‘the dread of consumption amongst the poor’ was ‘very much greater than the dread of notification’.^39^ Furthermore, interference with the future livelihood of the tuberculous patient was not an issue, because Sheffield's proposed law stated that ‘no provisions contained in any general or local Act of Parliament relating to infectious disease shall apply to tuberculosis of the lung’.^40^ This meant that patients could not be removed to hospital after notification (at least not without their consent). Sheffield did not operate a sanatorium of its own at this point.^41^

Another aspect of Sheffield's law concerned the domesticated nature of the actions taken after notification. There were two elements to this: domiciliary education and disinfection. The city made it apparent that it possessed the wherewithal to carry out disinfection measures. The LGB's Theodore Thomson argued the public, as consumers of health services, had every right to benefit from disinfection if their property rates were being used to pay physicians to notify their illnesses. Thomson used the language of citizenship to maintain that compulsory tuberculosis notification should not be granted ‘unless you have [disinfection] staff and appliances sufficient to deal with the problem’:

There is, in a case like this, as in very many other sanitary provisions, some interference with the liberty and possibly the comfort of the subject. I should not care to see a power given which conveyed that danger unless some adequate amount of benefit was to be obtained in return.^42^

Sheffield had modern disinfecting apparatus, adequate staff and an efficient administration to deal with the potential workload that compulsory notification would generate.^43^ The city's high-pressure steam disinfector had begun operation in 1888 and the annual number of houses disinfected leapt from around 30 in the mid-1880s to an average of more than one thousand by the early 1890s.^44^

As well as disinfecting homes and possessions, the second prong of Sheffield's policy of domestication was to give patients intensive education in the home. The position of Sheffield's Medical Officer of Health John Robertson was that public lectures and blanket leafleting of the community about the dangers of tuberculosis did not, and would not, have any lasting impact.^45^ In Sheffield and other cities with voluntary tuberculosis notification, patients and their carers already received verbal and written advice, in the domestic setting, about isolation, ventilation, disinfection and the disposal of sputum. A compulsory scheme meant that interventions could be targeted directly at people with active disease.

The House of Commons put compulsory notification of tuberculosis on trial in Sheffield for seven years. In 1910, the clauses were renewed for a further 10 years, as were those that had been given in the interim to the Lancashire towns of Bolton, Burnley and Oldham.^46^ These local acts were granted on the understanding that patients would not be compulsorily sequestered in sanatoria, that people would be educated about prevention, and that adequate facilities existed to disinfect patients' homes. All the local laws were superseded by a national act in 1912.

In this discourse about tuberculosis at the turn of the twentieth century, the spatiality of ‘home’ and ‘domestic’ was expressed as both site and scale. The home was naturalised as a location for intervention. Three points can be made about this. First, it is intriguing that this deliberative re-siting had to happen at all. Hospitals, workhouse infirmaries, lunatic asylums and pertinently in this case, tuberculosis sanatoria, were increasingly seen as the most appropriate sites of health care for many kinds of patients. Of course, a lot of sick people were still cared for at home, but institutionalisation destabilised this tradition, necessitating a restatement about the value of domiciliary care.^47^ Second, the domestication of state-sponsored disinfection activities provides a balance to the dominant view that responsibility for cleanliness in the home devolved solely onto housewives and mothers as popular knowledge about germs spread.^48^ Whilst not disputing this parallel trend, it is also plausible to argue that at the same time the state actively sought heightened obligations in the domestic arena. Furthermore, it was recognised that certain aspects of domestic architecture, materiality, organisation and social relations presented a risk to the health of tuberculosis patients and their relatives. The potential return on the mitigation of these risks was very high, which meant that the home had to be made into a viable site of effective regulation. Domestic space needed to be thought of and reproduced as a scale through which interventions could be imagined and implemented.

## Self-help and the diffusion of therapeutic space

The reluctance of Parliament to sanction the notification of tuberculosis partly helps to explain why, despite the mushrooming of private, public and voluntary sanatoria from the late nineteenth century, these institutions treated only a minority of people who had the disease in the early twentieth century. Worboys estimates there were 1500 sanatoria in-patient stays in 1900. By 1913 this had risen eightfold to 12,000 stays. This represented provision for less than 5% of all tuberculosis sufferers nationwide; or about 20% of patients with the active form of the disease.^49^

The ‘sanatorium benefit’ included in the National Insurance Act of 1911 stimulated more provision.^50^ From this date to 1920, the number of beds available for tuberculosis patients (excluding the Poor Law system) increased by about a factor of five, to almost 16,000.^51^ The term ‘sanatorium benefit’ was somewhat misleading, because it also provided funds for care beyond the walls of sanatoria, including home treatment. Most notably, local authorities were required to provide a tuberculosis dispensary that served as a diagnostic out-patient centre with welfare functions. The dispensary's tuberculosis officer and a team of nurses worked on a case-by-case basis in conjunction with the patient's GP to determine the most suitable treatment.^52^ Dispensary treatment rocketed after the National Insurance Act: there were 64 tuberculosis dispensaries in 1911; by 1920 there were 398.^53^

Debate about the effectiveness of home treatment seems to have intensified in the aftermath of the 1911 act. Some commentators, such as Oscar Holden, a tuberculosis officer with experience in Birmingham and Southampton, warned that the continuing risk of infection by advanced cases living at home was a serious danger to the wider community.^54^ Others were sceptical about the lack of moral support at home: anxious parents, relatives and friends made for bad advisors and it was impossible for them to be alert to all sorts of imprudent acts—kissing, visiting the pub, singing around the piano—that might endanger the patient and their family.^55^ On the other hand, some were convinced that many cases of tuberculosis could be successfully managed and cured at home, particularly with the additional level of monitoring offered by the dispensary system.^56^ Despite quite vehement support for both sides, there was no clear and convincing evidence about the benefits of either approach. An uneasy consensus emerged that sanatorium treatment was most suitable for early cases that were actively infectious, whereas home treatment was more appropriate for cases that were not currently infective or infective advanced cases that were beyond salvation.^57^

While precise details varied from place to place, a sanatorium ‘cure’ commonly included a combination of wholesome diet, exercise, graduated labour and plentiful fresh air.^58^ The involvement of the adult patient in deciding the parameters of their sanatorium treatment was important. Sanatoria staff tailored treatments to provide an individualised regime that the patient could manage him- or herself. Despite continual dispute about the regimen's therapeutic effectiveness, contemporaries nevertheless believed that sanatoria and the open-air movement—which transferred most activities including sleeping, eating, schooling and rest from indoors to out^59^—had the potential to ‘revolutionise social, municipal and national life’.^60^ Michael Worboys has gone so far as to suggest that the sanatoria-based ‘attempt at “cultural control” was of equal, if not greater, significance than the better-known Edwardian campaigns about motherhood and physical degeneration; indeed it may be artificial to separate them’.^61^

Historian Flurin Condrau's view is that the sanatorium has been mistakenly written about as a ‘façade behind which discipline, order and militaristic concepts of hygiene were used to control working-class sufferers’, and that more subtle historical interpretations should include the integration of patient agency into the therapeutic narrative.^62^ Whilst I broadly concur with Condrau—at the very least, the notion of ‘control’ might be more appropriately thought of as ‘regulation’—the predominant historical concern with the discrete site of the sanatorium potentially underestimates the wide-ranging influence of its therapeutic reach. A complementary focus on domestic preventive therapy—the design of which was drawn up in the sanatorium—clarifies how unsatisfactory the crude concept of ‘control’ really is. Furthermore, in the context of moral regulation, a rather complex set of practices of self-formation take shape in the domestic sphere that defy simple categorisation into projects articulated by the state and those performed by individuals on themselves. As Alan Hunt argues:

Projects of moral regulation and ethical self-formation frequently come together in the complex and varied forms of interaction between governing others and governing the self … a significant dimension of moral regulation projects is that they are projects directed at governing others while at the same time they result in self-governing effects … We need have no fear that the term moral regulation refers only to projects to impose external moral codes, even though history is replete with just such endeavours; moral regulation is often directed at inducing projects of self-formation, manifest in ubiquitous incitements to ‘self-control’.^63^

Self-control through tuberculosis education was popularised from the 1880s. The inculcation of both preventive and curative behaviours in the domestic sphere was adopted as a credible tactic because so few non-pauper tuberculosis sufferers were ever likely to be admitted to local institutions. Publicly- and privately-funded leaflet campaigns for tuberculosis control emerged in Britain in the mid-1880s, initially in Lancashire (notably Oldham and Manchester).^64^ These educational drives were conducted at the urban scale. Rhetorically, they identified transgressions in domestic and public space and codified appropriate forms of self-regulation: keep your home clean; ventilate it; disinfect furniture; maintain an unblocked chimney; destroy your sputum; practice open-air living; and stay away from close and crowded rooms, especially concert halls, theatres, and pubs. This is familiar historical ground. Yet, as we have seen in the case of Sheffield, some public health administrators grumbled about this dissipated technique of didactic intervention.^65^ Lifestyles and behaviours were simply too slow to change, if they did at all.

While leaflets and pamphlets had drawbacks, advice targeted directly at domiciled tuberculosis patients was also limited in that it was impossible for health visitors to monitor patients closely or reinforce the lessons on anything like a regular basis.^66^ Nor did the various local prevention schemes give detailed advice on how to approach the disease therapeutically. Some enterprising doctors identified this gap in the market and produced self-help manuals for the home-based tuberculosis patient. Medical self-help books have a long tradition.^67^ Those for consumptives that appeared at the beginning of the twentieth century were little different to many others that had gone before them in that they provided a fall-back mechanism for poor patients who were either unable or unwilling to avail themselves of medical attendance.^68^ However, tuberculosis self-help manuals were also directed at patients who had undergone a period of sanatorium treatment and, it was presumed, wanted it maintained in a domestic setting. Even then, a sanatorium stay was not necessarily a guarantee that the patient had been given the information needed to survive. In 1909, Sheffield's Medical Officer of Health, Henry Scurfield, bemoaned that ‘in many sanatoria, when the patient leaves he has not been educated’, even in some basic measures such as the disposal of sputum.^69^ These manuals were the product of a particular historical moment when sanatorium treatment was gaining credence as the most appropriate mode of therapy; at the same time, facilities did not exist to provide that treatment for all patients, and the chronic nature of the disease made the argument for long-term hospitalisation a tough one to make politically.

Essentially, the regime of home treatment replicated as far as possible that of the sanatorium. The key to success was a patient's self-knowledge of, and control over, his or her own body and its domestic surroundings. Bodily regulation was achieved by the constancy and repetition of physical conditioning: exercise, rest and diet were scrutinised as the patient undertook vigilant surveillance of her or his own temperature and weight. Patients were expected to eat, sleep and rest at prescribed times of the day and even to breathe in precise ways.^70^ Body temperature synchronised the eat–sleep–rest–exercise rhythms of home treatment; body weight was used to gauge the patient's progress.

Normal temperature and weight were achieved through strict control over dietary intake (the type and amount of food to be consumed), exercise (suitable forms of recreation were suggested), and the timing and extent of rest periods. Experts expressed differences of opinion about these aspects of the regime, differences that depended partly on the stage of the disease and partly on the individual patient; no two patients were the same.^71^ More important was the implementation of regularity and strict adherence to the set programme. Henry Hyslop Thomson wrote in his manual that: ‘The more closely one day conforms to another, and the more strictly the patient adheres to the routine of treatment, from day to day, and from week to week, the more beneficial and effective will the result be’.^72^ As such, disagreements over detail were overridden by the commonalities and the shared acknowledgement that some activities had a deeper moral purpose. Self-responsibility and strength of character were the key to concluding of a successful course of home treatment, be that complete cure or, more realistically, a prolonged period of capacity for work and the enjoyment of life. Noel Bardswell's guidance stressed that:

Character or temperament, as in all other things, is a very large factor in success. The irresponsible, the undisciplined, and the despondent have nothing like the same chance of recovery as the cautious, the level-headed optimist, and the man of purpose. It is well for the patient to recognise frankly, from the first, that the fight for life and health is to be a hard one, with the odds against him … the odds can be levelled up by learning the principles by which consumption may be cured, and resolutely adhering to them … the happy-go-lucky consumptive, though perhaps the shorter-lived, is a happier man that the discontented hypochondriac. He is certainly the more contented companion. The mid-course between these two extremes should be aimed at.^73^

These interlinking points about individual responsibility and determination constantly reiterated familiar tropes of self-help rhetoric. Thomson spoke of ‘personal effort’, ‘intelligent effort’, ‘constant effort’, ‘unswerving allegiance to the rules’ and ‘personal endeavour’. ‘In many cases’, wrote Thomson, ‘the consumptive holds in his hands the power of treating and curing himself … [he] … must intelligently order and supervise his whole method of living’.^74^ Bardswell was sure that there would be ‘little setbacks’ and the regime involved ‘much troublesome care and self sacrifice’.^75^ But the most successful patients, argued Henry Warren Crowe, will be ‘those who can form a resolution and steadfastly carry it out, and who are sufficiently master of their own surroundings’.^76^

Nurturing such characteristics was intrinsically linked to the prevention of immoral behaviours. Bardswell did not just emphasise the benefits of exercise when he advocated taking a walk around the local park each evening; he also believed that it staved off the temptation to stray into the pub and fritter money away on alcohol.^77^ According to Thomson, intemperance was the main predisposing cause of tuberculosis not because it corroded the liver of the habitual drinker, but because of ‘the conditions of life to which it gives rise. The children of the drunkard are badly clothed, indifferently fed and poorly housed, and readily fall prey to the ravages of tuberculosis’.^78^

As well as developing moral rectitude through bodily discipline, the patient had to comprehend the domestic conditions that mediated the risk of infection and predisposed their ‘deviation from the normal healthy standard’.^79^ Echoing the belief of Sheffield's public health officials, Thomson argued that the campaigns against poor ventilation, uncleanliness and inappropriate furnishings had made but a small dent on popular consciousness: there was still much ‘ignorance and mistaken views as to what constitutes a healthy home’.^80^ Crowe urged consumptives to study the direction of draughts through the house so that they might know where to position their bodies.^81^ No detail was ‘too trivial’ if the patient was to become ‘familiar with everything relating to himself and his surroundings’.^82^ The many constituents of domestic space—beds, furniture, windows, floor coverings and people—invariably were construed as problematic and risk-laden.

Awareness of the minutiae of domestic life was therefore exalted and this also had an explicit moral dimension. Intimate interactions with other family members represented an obstacle to the patient's progress. Of course, one of the main benefits of sanatorium treatment in the eyes of its promoters was isolation from meddlesome family and friends, which proved extremely difficult to replicate in the crowded domestic sphere.^83^ As with other infectious diseases, the patient was expected to take sole occupancy of a separate room in the house whenever possible.^84^ Even still, complete segregation was practically impossible, particularly for working men and women and housewives who moved in and around the house out of necessity.^85^ This feature of home treatment worried the writers of self-help manuals not only because the patient might continue to infect other family members, but also because the family could exert a corrupt influence on the stringent therapeutic regime.^86^ Consequently, the routine of the patient was aimed at restricting the likelihood of contact. Consumptives with active disease ‘should never be kissed on the lips’.^87^ Retiring early to bed not only delivered much-needed rest, but it also ‘removed the temptation to join the family circle in sitting up till a late hour’.^88^

Clearly, these texts were not just about health but also about morality. The emphasis in the tuberculosis self-help manuals on discipline, self-control, responsibility and the suppression of intimacy reflects the important role of character and morality in the daily life of the domiciled consumptive patient. These ideals had their high-water mark in the late Victorian period and have been associated with a type of ‘governmental self-formation’ through which authorities and experts explicitly seek to shape individuals' conduct.^89^ But the moment at which this tubercular self-help genre picks up was also the moment when the quest for ‘character’ shifted more towards subjectivity through self-discovery and the crafting of unique identity. Written from the standpoint of medical authority and expertise, yet emphasising individuality, self-quantification and bodily knowledge, these tuberculosis self-help manuals typify this transition. Indeed, it does not strike me as egregious to transplant to the early twentieth century Alan Hunt's argument that moral regulation today ‘is more likely to be found in the guise of self-help texts or the discourses of “addiction” and “recovery”. Yet such projects remain attempts at moral regulation in that they are concerned to effect changes in the conduct and ethical subjectivity of individuals’.^90^

## A material culture of consumption

In her book *Household Gods*, Deborah Cohen describes the complex, shifting relationship between domestic space and possessions, and what the two together signified about notions of the self at the turn of the twentieth century:

Possessions offered a lifeline for coming to terms with one's own identity in a society so much in flux. From its origins in the 1890s, the idea of ‘personality’ was fundamentally intertwined with the domestic interior. Character, an older conception of the self, connoted a moral state. Personality was about earned distinctiveness, performance, and display. No place was more of a stage for the turn of the century British than their homes—even if no one was else was watching.^91^

More of the intricacies and subtleties of the transition from ‘character’ to ‘personality’ can be teased out by looking at the consumer culture surrounding tuberculosis.^92^ It is worth stressing at this point that it was not as if the nurturing of moral character disappeared as a fundamental aspect of tuberculosis treatment. Well into the twentieth century, subsequent editions of the self-help manuals considered above (the third and, it transpired, final edition of Thomson's book was issued in 1928) were no less insistent on the importance of character than were the earlier versions.^93^ It continued to be common to think of consumptives as having character flaws that made them vulnerable to behavioural lapses which the ownership of, or contact with, certain things might help prevent. Nonetheless, it is difficult to resist the general thrust of Cohen's argument where the materiality of tuberculosis is concerned.

One conduit through which household commodities were marketed and sold to tuberculosis sufferers was a section of the *BJTB* entitled ‘Preparations and Appliances’. Medical and public health journals of the time carried direct advertising to supplement subscriptions and sustain print runs and circulation. Indeed, at the end of the nineteenth century, advertising constituted the largest revenue stream for prestigious medical journals.^94^ As with other publications, however, the *BJTB* also provided editorial information about commercial products that it thought might be of interest to its readers. There is no direct evidence to substantiate this claim, but it seems likely that the ‘Preparations and Appliances’ section of the journal involved a form of indirect advertising known as ‘puffing’ (now commonly known as ‘advertorials’); that is, the inclusion of an editorial item promoting the virtues of a product for which a payment was made by the advertiser. In the newspaper trade, a puff-piece commanded a higher rate than a regular advertisement because it carried the imprimatur of the newspaper itself.

By this date, the layout of direct advertising in most newspapers and professional journals gave the impression of a multitude of commodities jostling for space and attention. Advertisements were individually bordered and designed in an attempt to distinguish themselves from all the others on a congested page. In contrast to these sorts of pages at the front and back of the *BJTB*, however, ‘Preparations and Appliances’ was set in the journal's preferred typeface and column format. It looked no different to the main articles in the journal. In the *BJTB*'s running order, it frequently appeared between the book reviews and ‘Notes’, which was a segment that made readers aware of conferences they might attend, research they should keep up with, health resorts that could be visited, and tuberculosis institutions that needed patients (these topics help indicate the specialist readership of the journal, of which I can trace no firm evidence).^95^ In other words, ‘Preparations and Appliances’ was a sequential component in the *BJTB's* procession of tuberculosis commodification; an example of the way in which the power of private capital became institutionally entrenched.

Products appearing in ‘Preparations and Appliances’ were given a free promotional pass. Assertions claiming that a product ‘only needs to be known to be extensively used’,^96^ or that it ‘should gain an entrance to every home’,^97^ were just as common as the journal's assurances that it had tried and tested commodities extensively before recommending them.^98^ Though the journal claimed that it vetted these products, it is not known what filtering criteria (if any) were used to include or exclude particular commodities. The author(s) praising these products were not identified; such anonymity applied a veneer of objectivity. Yet manufacturers' artwork was reproduced faithfully and the promotional language was never critical.

Readers were directed either to retail outlets where goods could be purchased, or provided with the name and address of the manufacturer to buy direct. The journal's British audience of tuberculosis activists (sanatorium administrators, nurses, dispensary staff, and public health personnel), family doctors and patients was undoubtedly familiar with some form of purchasing-at-distance, either in response to advertisements in newspapers and magazines or through mail-order catalogues that were touted by agents. This type of consumer behaviour was not confined to the middle class. Networks of mail-order agents were also used by working-class people, partly because their origins were in the culture of working-class savings clubs.^99^ Mail-order shopping was boosted by the institution of the Royal Mail's parcel post in 1883. Just two years earlier the introduction of postal orders had given people without access to a cheque book a secure means of sending money. By World War One, £57 million worth of postal orders were issued and the General Post Office delivered more than 130 million packages each year. Much of this traffic was driven by the mail-order business.^100^ Journal writers at the *BJTB* and elsewhere who generated copy about commodities inserted themselves into this burgeoning mail-order trade, more or less acting like those catalogue agents who were working steadily in communities across the country.^101^ Though plying their powers of persuasion from a distance and in print, these publicists compiled and showcased inventories of potentially useful products and cajoled readers into contacting a manufacturer or retailer for further details, if not enticing them into an impulse purchase.

The motors of this consumer culture were rising incomes, the expansion of the middle classes—who, by 1901, represented 25% of the national population—and the ready availability of products through mass manufacture.^102^ The purchasing power of both the working and middle classes increased, but the latter spent a smaller proportion of their incomes on housing and necessities such as food and heat. When the middle class's disposable income was expended on filling homes with possessions, the explosion of acquisitive behaviour abraded Victorian notions of religious restraint, particularly given the indelible influence of domestic space over individual character. One solution to this dilemma, argue Cohen and other historians of domestic material culture, was to bestow possessions with moral qualities. Household goods conferred domestic propriety and decency. Appropriate, well-designed furnishings raised the moral tone, whereas inappropriate fixtures and fittings indicated deceit or ugliness that should not be tolerated: ‘By redefining consumption as a moral act, and the home as a foretaste of the heaven to come, the British middle classes sought to square material abundance with spiritual good’.^103^ The right sort of mass-produced possessions and their appropriate arrangement in domestic space denoted morality, spirituality, distinctiveness and personality.

I do not claim that the *BJTB*'s puff pieces tell us very much, if anything at all, about the motives and actions of tuberculous consumers. Rather, I want to suggest that ‘Preparations and Appliances’ was an interstice where a common tubercular ‘language’ of material consumption was fashioned, particularly for the middle-class patient who was more able to afford most of the goods on offer. The journal's readers were educated on how to visualise the domiciled tuberculosis patient. The things on display helped solidify the idea of the home-based patient in their minds. This language could be understood by sanatorium officials, public health activists, family doctors, patients and patient carers alike; not because it was specific or unique to the world of tuberculosis activism, but because it was ubiquitous.^104^ The pages of *BJTB* revelled in a technology of possession that sought to integrate tuberculosis patients into a contemporary consumer culture that everyone could recognise (if not partake of equally).^105^

A bewildering multitude of commodities were determined as ‘requisites’ of both the sanatoria *and* the ‘hygienic’ home. Some of these objects were explicitly medical, but their portability served to unify the regimen of these discrete spaces. The thermometer is a good example. The ‘Presto Thermometer’ purportedly overcame the deficiencies of most other thermometers, which could be slow acting, indistinctly marked and difficult to adjust, if not defective altogether. The Presto model had a scale marked for a range of 12 °C (94 °C–106 °C) instead of the customary 16 °C or 20 °C; these more generously-spaced fractional divisions were easier for the patient to read. Markings above the ‘normal’ temperature were indicated in red.^106^

**Fig. 1:**
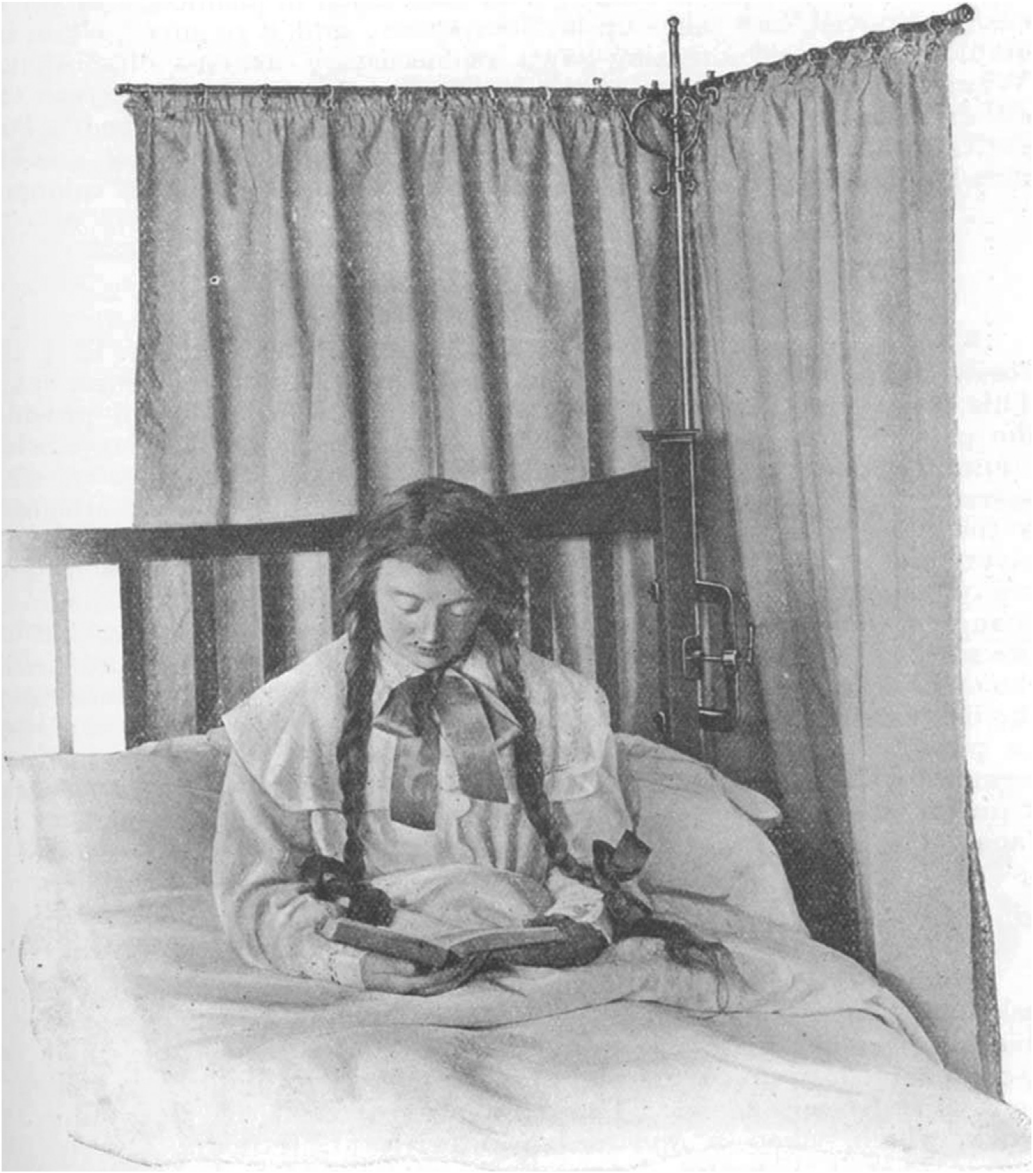
The ‘Epic’ patent bed-screen.

Source: Reprinted from: A sanatorium bed-screen, *British Journal of Tuberculosis* 8 (1914) 53, with permission from Elsevier.

Some items were specifically related to the practice of open-air living. Window tents made it possible to sleep close to an open window but protected the patient from the elements and the rest of the house from the bracing cold. Steel-framed awnings made from canvas were manufactured with the intention that the patient, lying in an extendable cot-like bed, would sleep with his or her head and shoulders completely outside the window frame.^107^ Significantly, bed screens and shelters were shown at the first Ideal Home Exhibition in 1908. A screen protected the patient from the wind and direct sunlight, as well as providing privacy. These devices sought to simplify the mechanics of sanatorium therapy for the domesticated patient: they were easily legible, readily manoeuvrable, and had manageable dimensions.^108^ One revealing aspect to the promotional images for many rest-related products is that patients tended to be reading (Fig. 1). Portrayal of an intellectually-stimulating behaviour shaped the common understanding of how a domiciled tuberculosis patient should look and what they should be doing. In this case, one possible visual chain message was that possession of a screen had a moral purpose because it allowed control of the immediate environment, which in turn facilitated character-building activities like reading.^109^

**Fig. 2:**
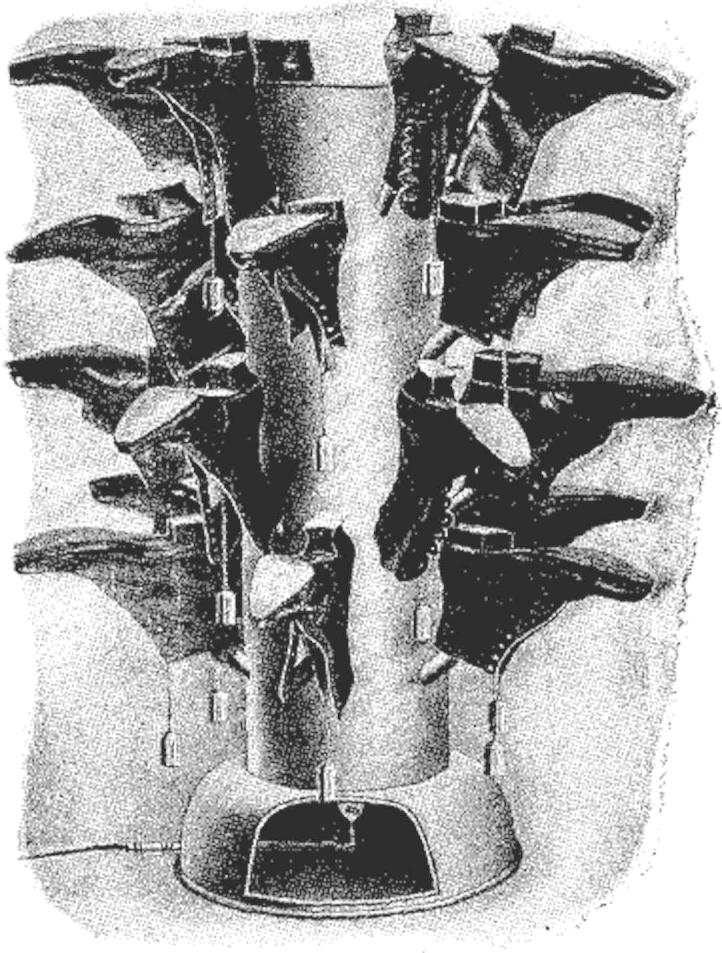
Hot air boot dryer.

Source: Reprinted from: A hot air boot dryer, *British Journal of Tuberculosis* 4 (1910) 138–139, with permission from Elsevier.

Keeping the home free from dirt was hard when practicing the open-air life. Anything that helped prevent mud from crossing the threshold was a boon. The ‘Major’ boot cleaner used revolving cocoa-fibre brush mats, steel wire and an underfoot scraper to remove dirt. A hot air boot dryer delivered a regulated current of warmth up a tree-shaped funnel (Fig. 2). This was particularly helpful in the rainy British climate. Shoes dried more quickly (but not so quickly so as to scorch and shrivel them) thus enabling frequent walks without the uncomfortable sogginess of damp leather.^110^ Given the iconic status tuberculosis activists granted to dust, the *BJTB* observed with some relief that because of contraptions such as these, ‘at last it would seem that homes may be kept free from dust all the year round’.^111^

**Fig. 3:**
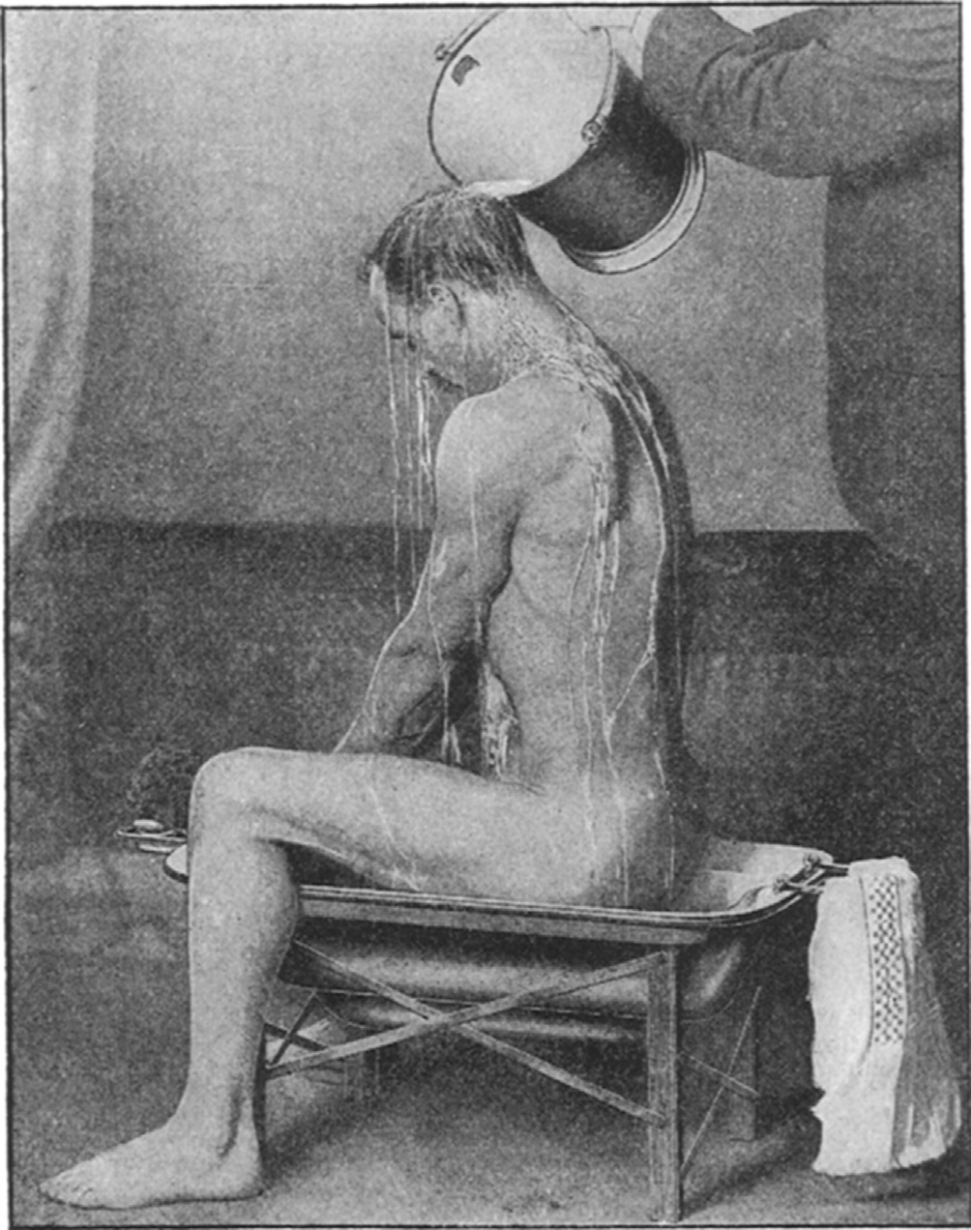
The Beb Bath in use.

Source: Reprinted from: The ‘Beb’ Bath, *British Journal of Tuberculosis* 3 (1909) 72, with permission from Elsevier.

Other types of equipment explicitly inculcated hygienic norms. The ‘Beb’ Bath, for example, could be used in the bedroom or on the sanatorium ward (Fig. 3). Moreover, it was marketed as affordable way of instilling bodily cleanliness, since it was on sale ‘at the specially low price of 13*s* 6*d* for the working classes’.^112^ How affordable these sorts of devices were is open to question. Thirteen shillings represented more than half of the average labourer's weekly earnings and a couple of shillings more than the average weekly wage for a working woman.^113^ Portable disinfection spray pumps and cleaning equipment that were relatively cheap also delivered the ‘gospel of germs’ into the home.^114^ Lightweight, with directional nozzles and long handles, these appliances could reach every surface and penetrate the darkest nooks and crannies.

**Fig. 4:**
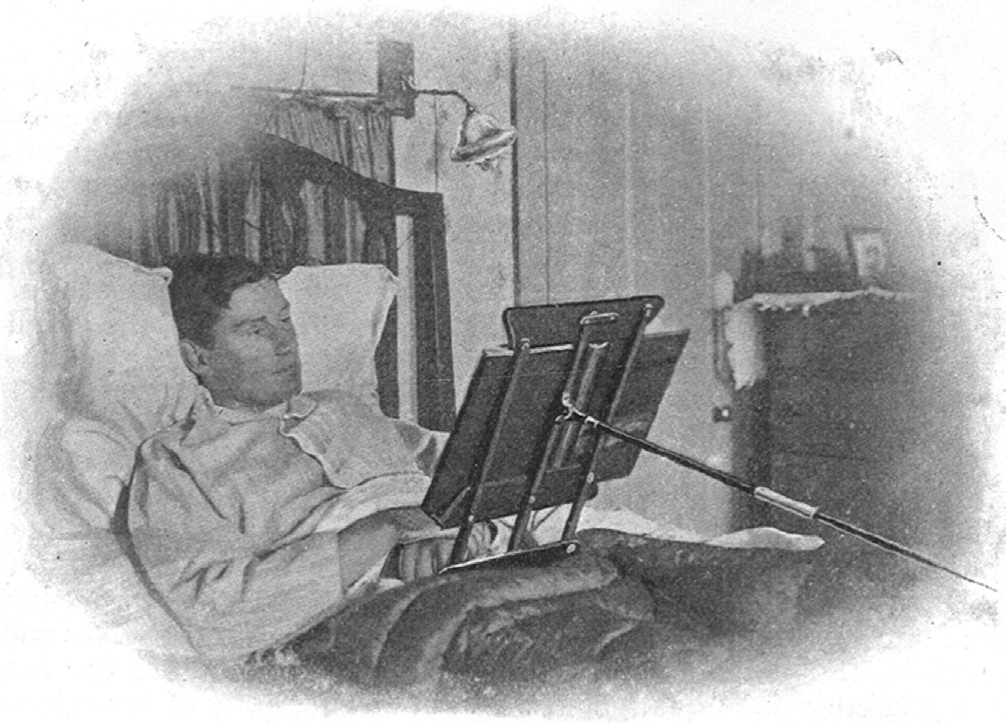
The ReferReader.

Source: Reprinted from: The ReferReader, *British Journal of Tuberculosis* 4 (1910) 57–58, with permission from Elsevier.

**Fig. 5:**
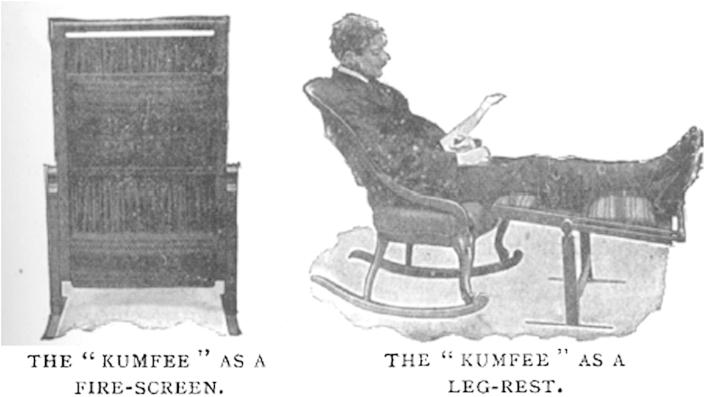
The ‘Kumfee’ in use as a fire-screen (left) and a leg-rest.

Source: Reprinted from: The ‘Kumfee’, *British Journal of Tuberculosis* 4 (1910) 211, with permission from Elsevier.

**Fig. 6:**
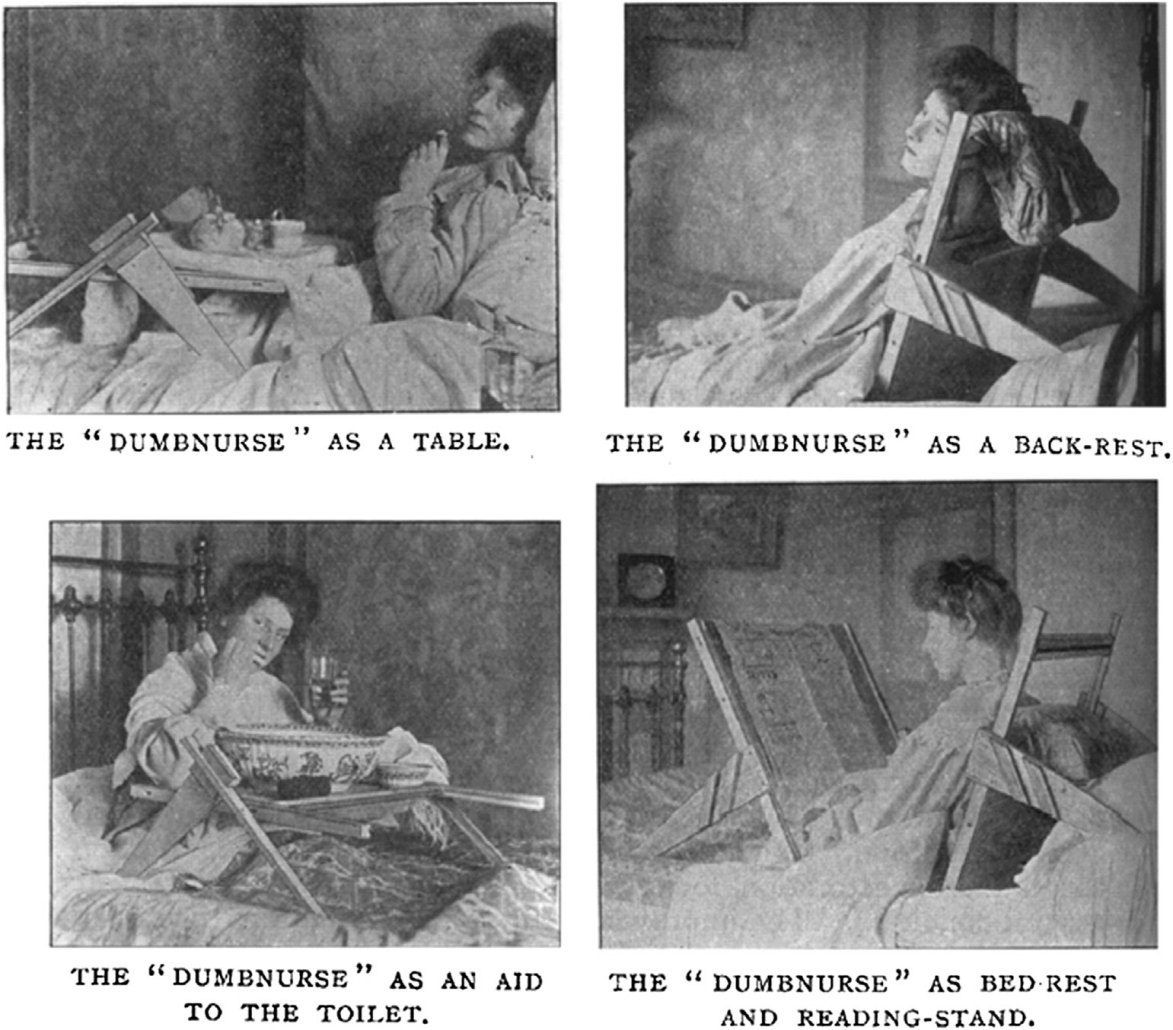
The various uses of the DumbNurse.

Source: Reprinted from: The ‘DumbNurse’, *British Journal of Tuberculosis* 6 (1912) 192, with permission from Elsevier.

As mentioned earlier, rest was a vital (if contested) feature of the sanatoria regime. Many commodities invited the home-based tuberculosis patient to become involved in middle-class leisure pursuits that complemented long periods of respite.^115^ Bed rests, foot rests and telescopic reading stands such as the ReferReader (Fig. 4) were presented as an entryway into the market of goods—some luxurious, others less so—that multiplied the contentment of modern life.^116^ The Kumfee, which doubled up as a leg-rest and fire-screen, was ‘an ingenious addition to the comforts of life which will be appreciated by invalids, luxury-lovers, and weary workers … appeals to both healthy and sick, and it is certainly an economiser of energy and an increaser of comfort’ (Fig. 5).^117^ The DumbNurse provided ‘the greatest comfort and convenience to patients, invalids, and, perhaps we might add, healthy luxury-lovers’.^118^ For the bed-ridden, the DumbNurse was a table, a backrest, and a reading stand (Fig. 6). Similarly, the ‘Axis’ portable bed table could be used for personal hygiene rituals such as washing and shaving, the serving of meals and as a table for writing, work or playing cards.^119^

This kind of multi-functionality was equated with sophistication which, in turn, indicated luxury. Designers of these products were responding to ideas about social relations as well as potential profitability in the market. Multiple designs of the same product were aimed at consumers who were stratified by sex, age, social class, and purchasing power. Product variation also served other aims. Not only did it anticipate an increase in sales, but it also spread the risk for a producer who was unsure about the market for a particular item. Variety promoted fashion by leaving older products behind. Such novelty was commensurate with the notion of consumer choice. The proliferation of options for what were essentially similar products was an important consideration for consumers who were keen to express their individuality through possessions.^120^

The novelty of design also applied to product naming. Manufacturers used portmanteau words (the blending of two words to create a new one, such as ReferReader and DumbNurse) and new spellings of old words (such as ‘Kumfee’) to differentiate their merchandise in a crowded market and to create product excitement. Each new version of a product represented yet another breakthrough or step forward. At the same time, the constant repetition of novelty and variety in media advertisements granted such developments the trajectory of predictability; rendering progress inevitable was a vital mechanism of modernity.^121^ So it was that every few months, readers of the *BJTB* could expect to marvel at yet another tranche of innovative products that would improve the lives of domesticated tuberculosis patients.

The examples highlighted here are merely representative. Space precludes consideration of further products such as clothes, heating appliances, telephone mouthpieces, even plasticine … the list is a very long one. Suffice to say, the *BJTB* lost no opportunity to enlighten its readers of the chance of aligning themselves with the materiality of modern living. Taken together, tapping the mass consumer market and succumbing to fetishised novelty heralded the expression of individuality that characterised the so-called ‘quest for personality’.^122^ The *BJTB* noted that these ‘numerous new inventions greatly facilitate the rational management of the sick and assist in the protection of the sound’.^123^ This was another way of saying that the putative presence of these commodities in domestic space had the capacity to soothe the sickly body, normalise tubercular life, and produce a watchful self-carer. Just like the self-help manuals written by Crowe, Thomson and Bardswell, the selection, arrangement and interaction with these objects conditioned subjective identity.^124^ They offered nothing less than a therapeutic toolbox for a materialised technology of the self that was deeply inflected with notions about the inherent morality of things.

## Conclusion

Towards the end of his *Advice to Consumptives*, Noel Bardswell encapsulated his recommendations with this little pearl of wisdom: ‘The best way, in short, of escaping consumption, is to live as if trying to cure it’.^125^ In this sense one can interpret the re-scaling and re-siting of the consumptive patient as essential ingredients in the emergence of a ‘preventive therapy’ for tuberculosis. Appropriation of domestic space transformed the sanatorium's ‘rules of health’ into ‘rules for living’. The very title of Crowe's manual—*Consumption: Treatment at Home and Rules for Living*—was unambiguous on this point.^126^

The term ‘preventive therapy’ also captures, but somewhat masks, what these experts sought to convey in terms of moral behaviour and to whom they were directing their efforts. The suppression of intimacy and avoidance of public houses made practical sense because it reduced exposure to infection.^127^ But hygienists consistently reiterated the deeper moral purpose of these several forms of abstinence that were the minimum preconditions for creating a domestic environment in which tuberculosis could not flourish. This message was buttressed by an openly acquisitive approach that denoted all sorts of worthiness; not least, many things promoted homely pastimes that distracted the patient from potentially immoral behaviours and towards wholesome interests.

It is impossible to deny the contradictions of the message and the medium. In the best traditions of nineteenth-century public health reform, an obvious intended target of these policy interventions, language, and images was the reckless moral behaviour of those working-class sufferers who constituted the main body of domiciled patients. Yet some of the risks (over-furnished homes for example) and some of the opportunities for recovery (such as the accumulation of appropriate possessions) were articulated through Victorian middle-class mores. They were the product of middle-class ideas about the typical patient's family and the presumed centrality of domesticity. Furthermore, the readership of the texts is not entirely clear. On the one hand, the knowledge promulgated in a specialist serial such as the *BJTB* was more likely to be passed on by doctors and activists verbally than fall into the hands of patients and carers directly. On the other hand, Crowe's *Consumption* was affordably priced at 1*s* for the working-class patient. He wanted GPs to buy the book who could then sell it on to their consumptive patients, thereby delivering the sanatorium regime into the home.^128^ Of course, the purchase and reading of a tuberculosis self-help manual was itself a moral form of consumerism, no matter the class of the patient. Who actually read the books, and to what extent the advice contained in them was acted on, are elusive questions at the moment. Perhaps these paradoxes and ambiguities are best read as the inevitable result of moral regulation exercised in a market of consumer goods that was not itself regulated very much.

Through the implementation of compulsory notification, disinfection, educational schemes, dispensary management and the home-based surveillance of patients and contacts, national and local government negotiated the legitimation and naturalisation of the home as a both a site and a scale of intervention. In this respect, these strategies can be seen as classics of their type: government administrations seeking ways to influence the self-conduct of individuals at a distance. It is important to emphasise that the formal power structures of the state prepared the ground on which the domiciliary self-regulation of tuberculosis patients gained traction.

This paper has shown that the production and reproduction of the domestic scale of moral regulation constituted a complex set of processes that were connected to wider social transformations in public health, consumer liberalism, subjectivity and governance of the self.^129^ State activities emerged from existing infectious disease policies; medical experts and activists worked inside and outside of formal government in ways that happened to complement the aims of the state; and, not least, the market was a crucial arena in which moral persuasion and regulation were played out.

## Footnotes

1 General sanitary measures implemented to combat environmental degradation and overcrowding in housing earlier in the nineteenth century probably had some effect on tuberculosis morbidity and mortality. The removal of many advanced cases to the workhouse may also have had an impact, however unintentional. For public health activity in Britain, see A. Hardy, *The Epidemic Streets: Infectious Disease and the Rise of Preventive Medicine, 1856–1900*, Oxford, 1993. The colonial imprint of tuberculosis is broached in M. Harrison and M. Worboys, A disease of civilisation, tuberculosis in Britain, Africa and India, 1900–1939, in: L. Marks, M. Worboys (Eds), *Migrants, Minorities, and Health: Historical and Contemporary Studies*, London, 1997, 93–124; M. Jones, Tuberculosis, housing, and the colonial state: Hong Kong, 1900–1950, *Modern Asian Studies* 37 (2003) 653–682. ‘Consumption’ as a term for respiratory tuberculosis had fallen away somewhat in British medical circles by the early twentieth century, though it was still in popular use. On ‘consumption’ as a wasting disease and its metaphorical association with eighteenth-century consumer society, see R. Porter, Consumption: disease of the consumer society?, in: J. Brewer, R. Porter (Eds), *Consumption and the World of Goods*, London, 1993, 58–81.

2 Today the term ‘preventive therapy’ is used in multiple medical contexts, though it is most commonly associated with tuberculosis. It comprises a combination of measures including isoniazid drug treatment to thwart the development of active disease, infection control, and intensified case finding.

3 L.H. Martin, H. Gutman and P.H. Hutton (Eds), *Technologies of the Self: A Seminar with Michel Foucault*, Amherst, 1988.

4 Much has been written about the artificiality of the private/public and home/work dichotomies and this paper is attuned to that. Contemporaries who wrote about and worked in tuberculosis therapy and prevention (and indeed, in other forms of infectious disease control) referred to the home as a discrete space and wondered how to gain access to it, configure it and manage the behaviours that went on inside it. For more on the inversion of privacy in the nineteenth century, see K. Chase and M.H. Levenson, *The Spectacle of Intimacy: A Public Life for the Victorian Family*, Princeton, N.J., 2000.

5 N. Rose, *Powers of Freedom: Reframing Political Thought*, Cambridge, 1999; A. Hunt, *Governing Morals: A Social History of Moral Regulation*, Cambridge, UK and New York, 1999, 198–199.

6 D. Cohen, *Household Gods: The British and their Possessions*, New Haven, 2006.

7 Hunt, *Governing Morals* (note 5), 7.

8 E. Swyngedouw, Scaled geographies: nature, place and the politics of scale, in: E.S. Sheppard, R.B. McMaster (Eds), *Scale and Geographic Inquiry: Nature, Society, and Method*, Malden, MA, 2004, 129–153, 132–133, 141.

9 M. White and A. Hunt, Citizenship: care of the self, character and personality, *Citizenship Studies* 4 (2000) 93–116.

10 For justification of the historical significance of the domestic scale, see S. Marston, The social construction of scale, *Progress in Human Geography* 24 (2000) 219–242; S. Marston, A long way from home: domesticating the social production of scale, in: E.S. Sheppard, R.B. McMaster (Eds), *Scale and Geographic Inquiry: Nature, Society, and Method*, Malden, MA, 2004, 170–191; A. Blunt and R. Dowling, *Home*, Abingdon, 2006, 26–29. For a recent overview of historical and contemporary work on ‘home’, see K. Brickell, ‘Mapping’ and ‘doing’ critical geographies of home, *Progress in Human Geography* 36 (2012) 225–244.

11 C. Hamlin, *Public Health and Social Justice in the Age of Chadwick: Britain, 1800–1854*, Cambridge, 1998; R.J. Morris, Governance: two centuries of urban growth, in: R.J. Morris, R.H. Trainor (Eds), *Urban Governance: Britain and Beyond Since 1750*, Aldershot, 2000, 1–14; S.R.S. Szreter, Economic growth, disruption, deprivation, disease, and death: on the importance of the politics of public health for development, *Population and Development Review* 23 (1997) 693–728; F. Driver, Moral geographies: social science and the urban environment in mid-nineteenth century England, *Transactions of the Institute of British Geographers* 13 (1988) 275–287.

12 C. Hamlin, Muddling in Bumbledom: on the enormity of large sanitary improvements in four British towns, 1855–85, *Victorian Studies* 32 (1988) 55–83.

13 It is worth emphasising here that the ‘local’ was far from an unproblematic administrative scale in the Victorian period.

14 A. Bashford, *Purity and Pollution: Gender, Embodiment and Victorian Medicine*, Basingstoke, 1998, 9; M. Poovey, *Making a Social Body: British Cultural Formation, 1830–1864*, Chicago, 1995, chapter 6; M. Levine-Clark, *Beyond the Reproductive Body: The Politics of Women's Health and Work in Early Victorian England*, Columbus, 2004, chapter 6.

15 G.K. Behlmer, *Friends of the Family: The English Home and its Guardians, 1850–1940*, Stanford, Calif., 1998, Introduction; L. Davidoff and C. Hall, *Family Fortunes: Men and Women of the English Middle Class, 1780–1850*, London and New York, 2002; D.O. Helly and S. Reverby, *Gendered Domains: Rethinking Public and Private in Women's History. Essays from the 7th Berkshire Conference on the History of Women*, Ithaca, N.Y., 1992.

16 P.K. Gilbert, *The Citizen's Body: Desire, Health, and the Social in Victorian England*, Columbus, 2007, chapter 2.

17 J. Harris, Between civic virtue and social Darwinism: the concept of the residuum, in: D. Englander, R. O'Day (Eds), *Retrieved Riches: Social Investigation in Britain, 1840–1914*, Aldershot, 1995, 67–87, 74–75; K. McClelland, England's greatness, the working man, in: C. Hall, K. McClelland and J. Rendall (Eds), *Defining the Victorian Nation: Class, Race, Gender and the Reform Act of 1867*, Cambridge, 2000, 71–118.

18 P. Joyce, *The Rule of Freedom: Liberalism and the Modern City*, London and New York, 2003, chapter 2; C. Otter, *The Victorian Eye: A Political History of Light and Vision in Britain, 1800–1910*, Chicago, 2008, chapter 3.

19 On governing ‘through’ the family, see M. Foucault, *Security, Territory, Population: Lectures at the Collège De France, 1977–1978*, Basingstoke and New York, 2007, chapter 4; J. Donzelot, *The Policing of Families*, New York, 1979, chapter 3.

20 For notification, see G. Mooney, Public health versus private practice: the contested development of compulsory notification of infectious disease in late-nineteenth-century Britain, *Bulletin of the History of Medicine* 73 (1999) 238–267; for isolation, G. Mooney, Infection and citizenship: (not) visiting isolation hospitals in mid-Victorian Britain, in: G. Mooney, J. Reinarz (Eds), *Permeable Walls: Historical Perspectives on Hospital and Asylum Visiting*, Amsterdam, 2009, 147–174. The concept of surveillance in public health is dealt with from different perspectives in D. Armstrong, The rise of surveillance medicine, *Sociology of Health and Illness* 17 (1995) 393–404, A.L. Fairchild et al., *Searching Eyes: Privacy, the State, and Disease Surveillance in America*, Berkeley and New York, 2007; B.S.A. Yeoh, *Contesting Space: Power Relations and the Urban Built Environment in Colonial Singapore*, Kuala Lumpur and New York, 1996, chapter 3.

21 P. Baldwin, *Contagion and the State in Europe, 1830–1930*, Cambridge, 1999; M. Worboys, *Spreading Germs: Disease Theories and Medical Practice in Britain, 1865–1900*, Cambridge, 2000.

22 On permissive legislation and localism, see A. Wohl, *Endangered Lives: Public Health in Victorian Britain*, London, 1983, 166–204. A representative example for notification is J. Tatham, On the compulsory notification of infectious diseases, *The Practitioner* 41 (1888) 472–480.

23 M. Worboys, Before McKeown: explaining the decline of tuberculosis in Britain, 1880–1930, in: F. Condrau, M. Worboys (Eds), *Tuberculosis Then and Now: Perspectives on the History of an Infectious Disease*, Montreal and Ithaca, 2010, 148–170.

24 P. Caldwell Smith, A discussion on isolation and disinfection: with special reference to the control of epidemics, *British Medical Journal* (1889) 455–456; R. Murray, *The Modern Householder: A Manual of Domestic Economy in all its Branches*, London and New York, 1872; E. Cleere, Victorian dust traps, in: W.A. Cohen, R. Johnson (Eds), *Filth: Dirt, Disgust, and Modern Life*, Minneapolis, 2005, 133–154.

25 J. Neiswander, *The Cosmopolitan Interior: Liberalism and the British Home 1870–1914*, New Haven and London, 2008, 57–81.

26 A. Carpenter, An address on domestic health, *British Medical Journal* 208 (1881) 1007–1009; see also Cleere, Victorian dust traps (note 24), 139.

27 W. Bardwell, *What a House Should Be, Versus Death in the House*, London, 1873; S.F. Murphy (Ed.), *Our Homes, and How to Make Them Healthy*, London and New York, 1883. Annmarie Adams discusses the ways in which doctors discredited architects' contribution to the healthiness of homes. See her *Architecture in the Family Way: Doctors, Houses, and Women 1870–1900*, Montreal and Kingston, 1996, 36–72. Architects such as Robert Edis and E.W. Godwin, Adams argues, were relegated to providing advice on decoration and furniture, precisely the aspects of middle-class homes that Carpenter was worried about. Also on Edis, see Neiswander, *The Cosmopolitan Interior* (note 25), chapter 3.

28 An American perspective can be found in G. Wright, *Moralism and the Model Home: Domestic Architecture and Cultural Conflict in Chicago 1873–1913*, Chicago, 1980, especially chapter 4.

29 Municipal governments did carry out cleansing operations, particularly during epidemics, but these usually focused on public spaces such as dirty streets, alleys and cesspits. See R.J. Morris, *Cholera 1832*, New York, 1976.

30 A.S. Delépine, Some experiments on the disinfection of rooms by gaseous formaldehyde, *Journal of State Medicine* (1898) 527–542.

31 The prevention of dust, *British Journal of Tuberculosis* 3 (1909) 224. For a broader statement, see A. Ransome, *Campaign Against Consumption: A Collection of Papers Relating to Tuberculosis*, Cambridge, 1915, 38–50. ‘Hygienists’ is the term used by historian Michael Worboys to characterise an approach of tuberculosis activists that emphasised health education and the provision of sanatoria. It was advocated by elite clinicians and was institutionalised through the work of the National Association for the Prevention of Consumption.

32 On the Victorian origins of ‘matter out of place’, see T. Crook, Putting matter in its right place: dirt, time and regeneration in mid-Victorian Britain, *Journal of Victorian Culture* 13 (2008) 200–222.

33 H. Timbrell Bulstrode, *Supplement to the Annual Report of the Medical Officer of Health to the Local Government Board 1905–06, on Sanatoria for Consumption and Other Aspects of the Tuberculosis Question* [Cd. 3657], London, 1907, 586; L. Bryder, *Below the Magic Mountain: A Social History of Tuberculosis in Twentieth-century Britain*, Oxford, 1988, 41–43.

34 The other towns were Bath, Bolton, Cardiff, Exeter, Liverpool, Manchester, Norwich, Scarborough, Sheffield, Stockport and Warrington: Timbrell Bulstrode, *Supplement to the Annual Report of the Medical Officer of Health to the Local Government Board 1905–06* (note 33), 586. On Bolton, see S. Robertson, ‘The terrible scourge of consumption is amongst us’: the Guild of Help, voluntarism and the problem of tuberculosis in Edwardian Bolton, in: K. Laybourn (Ed.), *Social Conditions, Status and Community, 1860–c.1920*, Stroud, 1997, 45–65.

35 Sheffield Archives, CA/608/(18), *Sheffield Corporation Bill 1903: Minutes of Evidence to House of Commons Select Committee*, London, 1903, Robertson, question 176, Newsholme, question 246. One reason why voluntary notification by GPs met with limited success was because the doctors feared that they would be liable to an action for damages by revealing a professional secret, especially if this resulted in a loss of earnings to the patient: J. Robertson, *Annual Report of the Medical Officer of Health for Sheffield, 1902*, Sheffield, 1903, 52.

36 Halifax Corporation was a victim of this policy in 1902. See Sheffield Archives, CA/608/(6), *Report of the Local Government Board on the Sheffield Corporation Bill, 1903*, London, 1903, 6; J.M. Eyler, *Sir Arthur Newsholme and State Medicine, 1885–1935*, Cambridge, 1997, 150.

37 Thomson had taken up his post in February 1888 and left in 1891 when he joined the medical department of the Local Government Board: Local Government Board, *Supplement to Twentieth Annual Report, Containing Report of Medical Officer, 1890* [C.6461], London, 1890–1891, x.

38 Sheffield Archives, *Report of the Local Government Board on the Sheffield Corporation Bill* (note 36), 5. The LGB did not object to voluntary notification, as long as the local authority paid a fee to the notifying practitioner: see Sheffield Archives, CA608, letters from Henry Sayer to Messrs Sherwood and Co., to the Secretary of the Local Government Board and to John Lithiby, Assistant Secretary of the Local Government Board; and letters from John Lithiby to Henry Sayer.

39 Sheffield Archives, *Sheffield Corporation Bill 1903* (note 35), Hall, questions 180, 212 and 216.

40 Robertson, *Annual Report of the Medical Officer of Health for Sheffield, 1902* (note 35), 53.

41 Some institutional space was provided locally by the poor law guardians, H. Scurfield, *Annual Report of the Medical Officer of Health for Sheffield, 1904*, Sheffield, 1905, 18. Later, the City granted £1000 per year to pay for some patients to go to sanatoria elsewhere.

42 Sheffield Archives, *Sheffield Corporation Bill 1903* (note 35), Thomson, question 282. See also questions 200–204.

43 Sheffield Archives, *Sheffield Corporation Bill 1903* (note 35), Boyce, questions 44–45.

44 F.W. Barry, *Report on Epidemic of Smallpox at Sheffield, 1887–88, by Doctor Barry, with Introduction by Medical Officer of Local Government Board* [C.5645], London, 1889, 8. On the operation of this Washington Lyons disinfector, see W.N. Twelvetrees, Disinfection: physical, chemical, and mechanical. Part II, *The Sanitary Record* NS 19 (1897) 505–507. The smallpox epidemic in 1888 prompted the disinfection of 3225 houses.

45 J. Robertson, *Special Report on the Prevalence of Tuberculosis and the Measures for its Prevention*, Sheffield, 1899, 10. See also Sheffield Archives, *Sheffield Corporation Bill 1903* (note 35), Hall, question 219.

46 *Bolton, Burnley, Oldham, and Sheffield (Local Government: Provisional Orders Confirmation)* (159), 1910.

47 D.N. Livingstone, *Putting Science in its Place: Geographies of Scientific Knowledge*, Chicago, 2003, 62–72; G. Mooney, Diagnostic spaces: workhouse, hospital and home in mid-Victorian London, *Social Science History* (2009) 357–390.

48 See N. Tomes, *The Gospel of Germs: Men, Women, and the Microbe in American Life*, Cambridge, Mass., 1998, chapter 6; Adams, *Architecture in the Family Way* (note 27), chapter 3.

49 M. Worboys, The sanatorium treatment for consumption in Britain, 1890–1914, in: J. Pickstone (Ed.), *Medical Innovations in Historical Perspective*, New York, 1992, 47–71, 64–65.

50 R.M. Leslie, State insurance and the machinery for the detection and treatment of tuberculosis, *British Journal of Tuberculosis* 5 (1911) 275–282; W.J. Howarth, Sanatorium benefit for tuberculous patients, and national insurance, *British Journal of Tuberculosis* 6 (1912) 242–244; H.H. Thomson, Insurance and sanatorium benefits, *British Journal of Tuberculosis* 6 (1912) 248–249; S. Davies and M. Fitzgerald, National insurance and the home treatment of consumptives, *British Journal of Tuberculosis* 6 (1912) 93–102; S. Vere Pearson, Observations on the working of ‘Sanatorium benefit’, *British Journal of Tuberculosis* 8 (1914) 8–15. See Bryder, *Below the Magic Mountain* (note 33), 36–41.

51 E.P. Hennock, *The Origin of the Welfare State in England and Germany, 1850–1914: Social Policies Compared*, Cambridge, 2007, 265. Many patients with tuberculosis who came under the poor law were treated at home.

52 The Paddington dispensary for tuberculosis: some general reflections, *The Lancet* 175 (1910) 867–868; D.J. Williamson, The future of dispensary and domiciliary management of tuberculosis, *British Journal of Tuberculosis* 7 (1913) 25–29. The dispensary in Sheffield also coordinated admissions to the city's tuberculosis hospitals and open-air schools.

53 Hennock, *The Origin of the Welfare State in England and Germany, 1850–1914* (note 51), 264–265; Bryder, *Below the Magic Mountain* (note 33), 33–36.

54 O. Holden, The danger of the domiciliary treatment of tuberculosis, *The Medical Officer* 11 (1914) 99–101; O. Holden, The domiciliary treatment of tuberculosis, *British Journal of Tuberculosis* 12 (1918) 81–85.

55 D.L. Cameron, Home treatment versus sanatorium: from the consumptive patient's point of view, *British Journal of Tuberculosis* 7 (1913) 99–101.

56 J. George, The bedroom of the consumptive, *British Journal of Tuberculosis* 10 (1916) 75–79; G. Jessel, Dispensary supervision in pulmonary tuberculosis, *The Lancet* 192 (1918) 894–895; see F.E. Wynne, Dispensary supervision in pulmonary tuberculosis, *The Lancet* 192 (1918) 680–681 for a counter-argument.

57 G.B. Dixon, The domiciliary treatment of tuberculous patients, *British Journal of Tuberculosis* 9 (1915) 19–22; F.C. Shrubsall, The selection of patients for sanatorium treatment, *Public Health* 26 (1912) 230–236; H. Clark, The organisation of scientific methods for the control of tuberculosis, *Public Health* 26 (1912) 332–337. For a spectrum of opinion across the early decades of the twentieth century, see: Discussion on the consumptive at the home, *Journal of the Royal Sanitary Institute* 27 (1906) 277–289; The care of the advanced consumptive, *British Journal of Tuberculosis* 13 (1919) 49–71. Any sort of reliable comparison of dispensary/home versus sanatorium treatment only began to appear in the 1920s. See G. Lissant Cox, Sanatorium treatment contrasted with home treatment: after-histories of 4067 cases, *British Journal of Tuberculosis* 17 (1923) 27–30.

58 Graduated labour was a feature of the British model of sanatorium treatment, whereas in Germany prolonged periods of rest were emphasised: F. Condrau, Urban tuberculosis patients and sanatorium treatment in the early twentieth century, in: A. Borsay, P. Shapely (Eds), *Medicine, Charity and Mutual Aid: The Consumption of Health and Welfare in Britain, c.1550–1950*, Aldershot, England and Burlington, VT, 2007, 183–205, 194–195.

59 Verandas, balconies, covered corridors, garden shelters, expansive areas of glass and the absence of structural supports were common ways that open-air life and sunlight were embedded in sanatoria architecture. See M. Campbell, What tuberculosis did for modernism: the influence of a curative environment on modernist design and architecture, *Medical History* 49 (2005) 463–488; A. Adams, K. Schwartzman and D. Theodore, Collapse and expand: architecture and tuberculosis therapy in Montreal, 1909, 1933, 1954, *Technology and Culture* 49 (2008) 908–942.

60 D.J. Chowry-Muthu, The sanatorium treatment of consumption: is it worthwhile? *British Medical Journal* 2 (1905) 46, quoted in Worboys, The sanatorium treatment for consumption in Britain (note 49), 58. On effectiveness, see Condrau, Urban tuberculosis patients and sanatorium treatment in the early twentieth century (note 58), 189–191. Bryder, *Below the Magic Mountain* (note 33), 49–53 contains some details on some of the purpose built structures for open-air living.

61 Worboys, The sanatorium treatment for consumption in Britain (note 49), 58–59.

62 Condrau, Urban tuberculosis patients and sanatorium treatment in the early twentieth century (note 58), 204–205. See also S. Craddock, Engendered/endangered: women, tuberculosis, and the project of citizenship, *Journal of Historical Geography* 27 (2001) 338–354; S. Kirby, Sputum and the scent of wallflowers: nursing in tuberculosis sanatoria, 1920–1970, *Social History of Medicine* 23 (2010) 602–620.

63 Hunt, *Governing Morals* (note 5), 16. Hunt's own discussion of moral regulation through ‘governmental self-formation’ and ‘ethical self-formation’ juxtaposes P. Corrigan, On moral regulation, *Sociological Review* 29 (1981) 313–337; P. Corrigan and D. Sayer, *The Great Arch: English State Formation as Cultural Revolution*, Oxford and New York, 1985; with M. Dean, ‘A social structure of many souls’: moral regulation, government, and self-formation, *Canadian Journal of Sociology* 19 (1994) 145–168. Hunt concludes that the commonalities between the two positions are more significant than the differences.

64 Oldham's campaign was funded by the local state. Manchester's was underwritten by the Manchester and Salford Sanitary Association, a private organisation whose membership was populated by public appointees such as poor law medical officers and medical officers of health. See J. Niven, On municipal administrative measures in connection with tuberculosis, *Tuberculosis. The Journal of the National Association for the Prevention of Consumption and Other Forms of Tuberculosis* 1 (1900) 185–196; Ransome, *Campaign Against Consumption* (note 31), 53. The gendered rhetoric of these educative materials tended to be implicit (at least compared to the explicitly gendered discourses of maternalism and physical degeneration).

65 Individualistic modes of explaining and tackling disease in the context of tuberculosis are discussed in G. Kearns, Tuberculosis and the medicalisation of British society, 1880–1920, in: J.H. Woodward, R. Jütte (Eds), *Coping with Sickness: Historical Aspects of Health Care in a European Perspective*, Sheffield, 1995, 147–170.

66 For an example of the work of tuberculosis nurses in an American context, S. Craddock, *City of Plagues: Disease, Poverty and Deviance in San Francisco*, Minneapolis and London, 2000, 222–229. Craddock draws attention to the advice nurses gave about the organisation of interior domestic space and cleanliness.

67 C.E. Rosenberg, Health in the home: a tradition of print and practice, in: C.E. Rosenberg (Ed.), *Right Living: An Anglo-American Tradition of Self-Help Medicine and Hygiene*, Baltimore, 2003, 1–20.

68 Some articles on home treatment appeared in the National Association for the Prevention of Consumption's short-lived journal *Tuberculosis*. A representative example is M.S. Paterson and F.C. Shrubsall, Hints for the consumptive at home, *Tuberculosis. The Journal of the National Association for the Prevention of Consumption and Other Forms of Tuberculosis* 4 (1906) 140–145.

69 H. Scurfield, *Annual Report of the Medical Officer of Health for Sheffield, 1909*, Sheffield, 1910, xiii.

70 H.W. Crowe, *Consumption: Treatment at Home and Rules for Living*, Bristol, 1906, 31–32. Crowe included an appendix on breathing exercises.

71 M.S. Paterson and F.C. Shrubsall, The home treatment of the consumptive invalid, *Tuberculosis. The Journal of the National Association for the Prevention of Consumption and Other Forms of Tuberculosis* 4 (1907) 178–184, 184.

72 H.H. Thomson, *Consumption, its Prevention and Home Treatment: A Guide for the Use of Patients*, London, 1911, 56. In 1911, Thomson was Medical Superintendent of the Liverpool Sanatorium at Frodsham. He later acquired a Diploma in Public Health from Liverpool University and became County Medical Officer of Health, School Medical Officer of Health and Tuberculosis Officer for Hertfordshire.

73 N.D. Bardswell, *Advice to Consumptives: Home Treatment, After-care and Prevention*, London, 1910, 74. Bardswell was the well-respected Medical Superintendent of the King Edward VII Sanatorium in Sussex.

74 Thomson, *Consumption, its Prevention and Home Treatment* (note 72), 28, 30, and 31.

75 Bardswell, *Advice to Consumptives* (note 73), 140; Bryder, *Below the Magic Mountain* (note 33), 223.

76 Crowe, *Consumption* (note 70), 7. Crowe was a practicing general practitioner when he wrote his manual. He later founded the Charterhouse Rheumatism Clinic in London in 1928 and joined the Empire Rheumatism Council. See D. Cantor, The aches of industry: philanthropy and rheumatism in inter-war Britain, in: J. Barry, C. Jones (Eds), *Medicine and Charity before the Welfare State*, London and New York, 1991, 225–245, 230.

77 Bardswell, *Advice to Consumptives* (note 73), 140.

78 Thomson, *Consumption, its Prevention and Home Treatment* (note 72), 13.

79 Thomson, *Consumption, its Prevention and Home Treatment* (note 72), 11. Occupational stress and over-work were warned against, but the improbability of changing jobs as a viable therapeutic strategy was acknowledged.

80 Thomson, *Consumption, its Prevention and Home Treatment* (note 72), 12. It must be said that bacteriological discoveries about the presence of tubercle bacilli in dried sputum propelled the significance of dust and the movement of fresh air to new heights. See Tomes, *The Gospel of Germs* (note 48).

81 Crowe, *Consumption* (note 70), 13.

82 Thomson, *Consumption, its Prevention and Home Treatment* (note 72), 46.

83 S.S. Holton, To live ‘through one's own powers’: British medicine, tuberculosis and ‘invalidism’ in the life of Alice Clark (1874–1934), *Journal of Women's History* 11 (1999) 75–96, 86.

84 Patients lucky enough to have a garden were encouraged to build a shelter: Thomson, *Consumption, its Prevention and Home Treatment* (note 72), 37. Some councils and sanatoria lent shelters out to patients after they were discharged: Bryder, *Below the Magic Mountain* (note 33), 224–226.

85 According to Newsholme, this was especially true for poor patients: A. Newsholme, Four and a half years' experience of the voluntary notification of pulmonary tuberculosis, *Journal of the Sanitary Institute* 24 (1903) 253–260, 254–255.

86 Crowe, *Consumption* (note 70), 4.

87 Thomson, *Consumption, its Prevention and Home Treatment* (note 72), 23.

88 Thomson, *Consumption, its Prevention and Home Treatment* (note 72), 55.

89 Hunt, *Governing Morals* (note 5), 16–18, 157. I am aware that this emphasis on auto-regulation and self-government downplays the fact that the main caregivers in the home were in most circumstances women. In fact, these self-help texts rarely mentioned the role of a caregiver and the tuberculosis sufferer was either masculinised or referred to in gender-neutral terms as the ‘patient’ or the ‘consumptive’. It is perhaps instructive here (if not directly comparable) that much recent research on the geography of caregiving has highlighted the neoliberal devaluation and depoliticisation of female care work in the privacy of the home. For two broad overviews, see J. Popke, Geography and ethics: everyday mediations through care and consumption, *Progress in Human Geography* 30 (2006) 504–512; V. Lawson, Geographies of care and responsibility, *Annals of the Association of American Geographers* 97 (2007) 1–11.

90 Hunt, *Governing Morals* (note 5), 17.

91 Cohen, *Household Gods* (note 6), xii. It was a contradiction that such expressions of individuality were purchased at the cost of conforming to notions and standards of taste shaped outside domestic space: A. Forty, *Objects of Desire: Design and Society Since 1750*, New York, N.Y., 1992, pbk Edition, 94. Cohen's ideas bear comparison to anthropologist Daniel Miller's arguments about materialism, morality and the possibility of humanity: D. Miller, The poverty of morality, *Journal of Consumer Culture* 1 (2001) 225–243, especially 226–233.

92 For the early twentieth century, Judith Neiswander traces a turn towards imperial and nationalistic fervour in interior decoration. I do not necessarily see this as being exclusive of the shift from ‘character’ to ‘personality’ detected by other scholars; connections and ruptures between the two strands deserve further exploration, not to mention the implicit shifts in scale. Neiswander, *The Cosmopolitan Interior* (note 25), chapter 6. For more on character formation and the morality of interior furnishings and decoration, see J. Jennings, Controlling passion: the turn-of-the-century wallpaper dilemma, *Winterthur Portfolio* 31 (1996) 243–264. On the transition to ‘personality’ in the American context, see B. Gordon, Woman's domestic body: the conceptual conflation of women and interiors in the industrial age, *Winterthur Portfolio* 31 (1996) 281–301.

93 Crowe's book ran to three editions in Britain, two in Canada and an edition that was adapted for the Indian market in 1917. The first edition of Thomson's guide was published by Oxford University Press in 1911. The third and last edition in 1928 was published by Milford and the disease in the book's title had changed from *Consumption* to *Tuberculosis*. The first and second editions of Bardswell's book came out in 1910 and 1920 respectively.

94 P.W.J. Bartrip, *Mirror of Medicine: A History of the British Medical Journal*, Oxford, 1990.

95 T. Nevett, Advertising and editorial integrity in the nineteenth century, in: M. Harris, A.J. Lee (Eds), *The Press in English Society from the Seventeenth to Nineteenth Centuries*, Rutherford, 1986, 154–164, 154–159.

96 Preparations and appliances, *British Journal of Tuberculosis* 1 (1907) 92–101, 92.

97 Foot-rests, *British Journal of Tuberculosis* 6 (1912) 63–64, 65.

98 Sanitary scraper and mat, *British Journal of Tuberculosis* 3 (1909) 75–75. *Baby's World*, founded in 1910, rejected advertisements when the products did not live up to the claims made for them: see L.G. Gurjeva, Child health, commerce and family values: the domestic production of the middle class in late-nineteenth and early-twentieth century Britain, in: M. Gijswijt-Hofstra, H. Marland (Eds), *Cultures of Child Health in Britain and the Netherlands in the Twentieth Century*, Amsterdam, 2003, 103–125, 108.

99 R. Coopey and D. Porter, Agency mail order in Britain, c. 1900–2000: spare-time agents and their customers, in: J. Benson, L. Ugolini (Eds), *A Nation of Shopkeepers: Five Centuries of British Retailing*, London and New York, 2003, 226–248; R. Coopey, S. O'Connell and D. Porter, *Mail Order Retailing in Britain: A Business and Social History*, Oxford, 2005, especially 13–38.

100 M.J. Daunton, *Royal Mail: The Post Office since 1840*, London and Dover, N.H., 1985, 57–64; C.R. Perry, *The Victorian Post Office: The Growth of a Bureaucracy*, London, 1992, 226–227.

101 Coopey and Porter, Agency mail order in Britain, c. 1900–2000 (note 99).

102 J. Benson, *The Rise of Consumer Society in Britain, 1880–1980*, London and New York, 1994, 23–26.

103 Cohen, *Household Gods* (note 6), 30. See also T. Logan, *The Victorian Parlour*, Cambridge and New York, 2001.

104 Adapting terms from C. Breward, *The Hidden Consumer: Masculinities, Fashion and City Life 1860–1914*, Manchester and New York, 1999, 160; L. Ugolini, Men, masculinities, and menswear advertising, c. 1890–1914, in: J. Benson, L. Ugolini (Eds), *A Nation of Shopkeepers: Five Centuries of British Retailing*, London and New York, 2003, 80–104, 81.

105 ‘Technology of possession’ is borrowed from A.M. Cronin, Rags and refuse: the newspaper, empire, and nineteenth century commodity culture, *Cultural Studies* 20 (2006) 574–598, 583.

106 The image that accompanied the description of the Presto thermometer contradicted the text and showed a 16 °C scale: The saranac thermometer, *British Journal of Tuberculosis* 12 (1918) 93–94.

107 T.S. Carrington, The domiciliary management of consumptives, *British Journal of Tuberculosis* 7 (1913) 139–151. In other situations (particularly in poorer households), patients and carers jerry-rigged household fixtures and fittings into raised platforms that brought them closer to an open window. See the contraption assembled by a dispensary nurse in Edinburgh using a sink top, wooden plank and mattress, pictured in Campbell, What tuberculosis did for modernism (note 59), 476.

108 A bed-screen and shelter, *British Journal of Tuberculosis* 3 (1909) 75–76. This screen was priced at 8*s* 6*d*.

109 A sanatorium bed-screen, *British Journal of Tuberculosis* 8 (1914) 53–54. This screen was also recommended for patients in their own homes.

110 Requisites for the sanatorium and the hygienic home, *British Journal of Tuberculosis* 4 (1910) 60–61; A hot air boot dryer, *British Journal of Tuberculosis* 4 (1910) 138–139.

111 The ‘Boreas’ sweeper, *British Journal of Tuberculosis* 3 (1909) 150–151.

112 A new form of bath, *British Journal of Tuberculosis* 3 (1909) 72–73.

113 Weekly wages from N. Crafts, I. Gazeley and A. Newell, *Work and Pay in 20th Century Britain*, Oxford, 2007, 67, Table 3.8.

114 The Perfection Sprayer was made by Newton, Chambers and Co., who already manufactured *Izal* disinfectant, A sprayer for disinfectants, *British Journal of Tuberculosis* 3 (1909) 220–220. The sprayer was comparatively affordable; the three models cost 4*s*, 4*s* 9*d*, and 7*s* 6*d*.

115 The ‘cure chair’ is discussed at length in M. Campbell, From cure chair to ‘chaise longue’: medical treatment and the form of the modern recliner, *Journal of Design History* 12 (1999) 327–343.

116 See also: A self-adjustable bed-rest, *British Journal of Tuberculosis* 3 (1909) 295; A pillow-sling and bed-table, *British Journal of Tuberculosis* 3 (1909) 73; A folding leg-rest, *British Journal of Tuberculosis* 4 (1910) 214; The ‘Lansdown’ bed-rest, *British Journal of Tuberculosis* 8 (1914) 55; The ‘Lianrite’, *British Journal of Tuberculosis* 9 (1915) 158–159.

117 The ‘Kumfee’, *British Journal of Tuberculosis* 4 (1910) 211, retailed at 25*s*.

118 The ‘DumbNurse’, *British Journal of Tuberculosis* 6 (1912) 191–192. The DumbNurse was priced at 15*s*.

119 A combined portable bed-table and washing-stand, *British Journal of Tuberculosis* 12 (1918) 138–141.

120 Forty, *Objects of Desire* (note 91); T. Richards, *The Commodity Culture of Victorian England: Advertising and Spectacle, 1851–1914*, Stanford, Calif., 1990, 7.

121 Cronin, Rags and refuse (note 105).

122 White and Hunt, Citizenship (note 9), 105.

123 Appliances for the patient, *British Journal of Tuberculosis* 4 (1910) 57–60, 57–58.

124 See D. Fuss, *The Sense of an Interior: Four Writers and the Rooms that Shaped Them*, New York and London, 2004, 15–16.

125 Bardswell, *Advice to Consumptives* (note 73), 137.

126 It is worth pointing out here that institutions also expropriated the form and content of domestic space as a therapeutic strategy: M. Guyatt, A semblance of home: mental asylum interiors, 1880–1914, in: S. McKellar, P. Sparke (Eds), *Interior Design and Identity*, Manchester, 2004, 48–71.

127 J. Niven, A discussion on the relation of phthisis to factory and workshop conditions, *British Medical Journal* 2 (1902) 761–766, was one of the first to suggest that the common practice of spitting on the floors of public houses made them dangerous places.

128 Crowe, *Consumption* (note 70), 4. This was the price of the first edition of Crowe's book in 1906. Bardswell's cost 1*s* 6*d*, Thomson's 2*s*.

129 Indeed, geographers of contemporary care have begun to look more closely at the home as a complex space in which, amongst other things, ‘numerous state institutions, practices and procedures are enacted and reproduced on a daily basis’, K. England, Home, work and the shifting geographies of care, *Ethics, Place and Environment* 13 (2010) 131–150, 135. See also M. Brown, Hospice and the spatial paradoxes of terminal care, *Environment and Planning A* 35 (2003) 833–851.

